# MIC distribution analysis identifies differences in AMR between population sub-groups

**DOI:** 10.12688/wellcomeopenres.21269.1

**Published:** 2024-05-09

**Authors:** Jacob Wildfire, Naomi R. Waterlow, Alastair Clements, Naomi M. Fuller, Gwen M. Knight

**Affiliations:** 1 Department of Infectious Disease Epidemiology, Faculty of Epidemiology and Population Health, London School of Hygiene and Tropical Medicine, London, London, WC1E 7HT, UK; 2 Centre for Mathematical Modelling of Infectious Diseases, London School of Hygiene & Tropical Medicine, London, WC1E 7HT, UK; 3 Institute for Infection and Immunity, St George's, University of London, London, London, SW17 0RE, UK

**Keywords:** Antimicrobial Resistance, AMR Surveillance, MIC distributions

## Abstract

**Background:**

Phenotypic data, such as the minimum inhibitory concentrations (MICs) of bacterial isolates from clinical samples, are widely available through routine surveillance. MIC distributions inform antibiotic dosing in clinical care by determining cutoffs to define isolates as susceptible or resistant. However, differences in MIC distributions between patient sub-populations could indicate strain variation and hence differences in transmission, infection, or selection.

**Methods:**

The Vivli AMR register contains a wealth of MIC and metadata for a vast range of bacteria-antibiotic combinations. Using a generalisable methodology followed by multivariate regression, we explored MIC distribution variations across 4 bacteria, covering 7,135,070 samples, by key population sub-groups such as age, sex and infection type, and over time.

**Results:**

We found clear differences between MIC distributions across various patient sub-groups for a subset of bacteria-antibiotic pairings. For example, within *Staphylococcus aureus*, MIC distributions by age group and infection site displayed clear trends, especially for levofloxacin with higher resistance levels in older age groups (odds of 2.17 in those aged 85+ compared to 19–64), which appeared more often in men. This trend could reflect greater use of fluoroquinolones in adults than children but also reveals an increasing MIC level with age, suggesting either transmission differences or accumulation of resistance effects. We also observed high variations by WHO region, and over time, with the latter likely linked to changes in surveillance.

**Conclusions:**

We found that MIC distributions can be used to identify differences in AMR levels between population sub-groups. Our methodology could be used more widely to unveil hidden transmission sources and effects of antibiotic use in different patient sub-groups, highlighting opportunities to improve stewardship programmes and interventions, particularly at local scales.

## Plain Language Summary

Resistance of bacteria to antibiotics is a global problem and causes millions of deaths every year. How resistant an organism is to an antibiotic can be measured very easily and cheaply and can potentially provide a lot of information about bacterial evolution and how to use the right antibiotics to treat infections.

We took multiple large, global collections of these measurements and combined them together. We then took this large dataset, and looked at whether any differences in the degree of resistance could be seen when you separated the bacteria by the background of the patient they came from. In other words, we looked at whether certain groups of patients had more or less resistant bacteria.

For some very important bacterial species, we found that age played a strong role, with some bacteria from older people having more resistance against some antibiotics. Also, generally men had infections with bacteria with more resistance. The type of infection was also important, as was the region of the world that the patient was from, with South-East Asia generally having more risk of higher resistance. These results highlight that we can use this data to discover more subtle differences in the bacteria causing infections that different patients suffer from. This could help us to change how we use antibiotics, so that we can maximise their effectiveness for longer.

Whilst these results were very interesting, the main thing that we hope to highlight is that this method could be used effectively in local hospitals, where resistance data is collected routinely and often, to try and help doctors to understand AMR in their settings, intervene to prevent spread and better prescribe antibiotics day-to-day.

## Introduction

The global burden of antimicrobial resistance (AMR) is growing, yet there remain huge unknowns concerning the drivers and dynamics of its spread. Due to a lack of understanding of whether antimicrobial use or transmission drives more AMR infections, we cannot optimally target interventions, with many stewardship interventions failing to impact AMR rates ^1– 3^. Evidence suggests that locally targeted interventions using local data to implement cost-effective solutions are urgently required, particularly in low-resource settings ^4^. 

Minimum inhibitory concentration (MIC), defined as the lowest concentration of a chemical, usually a drug, which prevents visible *in vitro* growth of bacteria ^5, 6^, is one of the most routinely collected types of AMR surveillance data globally. Its primary use is to determine susceptibility phenotype (susceptible or resistant), used for clinical purposes and in AMR surveillance studies. MIC data, however, contains more information that is lost in this conversion to discrete categories. Also, MIC is an absolute measurement (though there can be differences by measurement assay) and therefore allows for greater comparison over time than susceptibility phenotype, which is dependent on ever-developing break-point definitions ^7– 9^.

To our knowledge, historical exploration of MIC data has been limited to examination of MIC “creep” (changes over time e.g. 10, 11) and country-level comparisons ^12^. One database, Pfizer’s Antimicrobial Testing Leadership and Surveillance (ATLAS) programme ^13^, contains 17 years of cumulative global MIC and rich metadata. Catalán *et al.* (2022) used the ATLAS database to demonstrate that global changes in MICs for particular bacteria-drug combinations over time can be predicted using the dataset, identifying some gradual global increases (MIC creep) ^14^. Similarly, Kenyon *et al.* (2019) used historical *Neisseria gonorrhoea* MIC distribution data from five countries to track the replacement of susceptible strains by increasingly resistant ones, raising questions about whether resistance is reversible ^12^. These studies highlight the value of MIC analyses, in particular with ATLAS, and how spatiotemporal analyses of MIC distributions can be used to fill knowledge gaps regarding AMR evolutionary dynamics.

We suggest here a new analysis framework that explores how distributions of MIC values could be used to uncover resistance evolution and transmission between patient populations and hence improve intervention targeting. These MIC distribution comparisons could be used in many settings to explore more subtle and local differences in resistance evolution as MIC data is widely, rapidly and cheaply available. Differences in MIC distributions from bacteria isolated from different patient groups could suggest different transmission routes or more rapid resistance evolution in the infecting strains. This could be driven by known differences in antibiotic effects or exposures between patient groups and could be used to measure the impact of stewardship or infection prevention measures that target groups differently. For example, our research group has found distinct resistance prevalence patterns by age and sex for different bacteria-antibiotic combinations at the national level ^15^ that could be linked to a wide range of factors such as antibiotic exposure, contact patterns and infection incidence. Known differences exist in the strains that cause different infection syndromes, *e.g.* lung and gastrointestinal infections ^16, 17^.

As part of the Vivli AMR Surveillance Open Data Re-Use Data Challenge, we explored MIC distribution variation within a novel stratification framework to explore whether we can detect substantial differences between sub-groups of isolates, grouped by patient age and sex, infection site, geographical region and over time.

## Methods

### Databases and cleaning

We explored the six available databases in the Vivli AMR Register ^18^ in August 2023 and incorporated their data in our analyses if they included (a) MIC values and (b) metadata on age and sex. This excluded the DREAM and SIDERO-WT databases. We combined the other four databases: ATLAS, SOAR 201818, KEYSTONE, and GEARS to obtain a total of 24,385,403 susceptibility results. Of these, 324,275 results were missing age information (1.3%), 248,583 were missing sex (1.02%) and 1180 had sex classified as an “N” (0.004%), which we further excluded.

The MIC data was cleaned by excluding all alpha-numeric values (often appearing to refer to genetic determinants, e.g. “OXA-“). This removed 45% of the susceptibility results (13,180,594 results remaining). Values with a qualifier (e.g. < or ≤) were set at the numeric value in the qualifier (e.g. <8 was set to 8).

Data on “body site” or “source” was grouped from 170 unique locations into five key “infection site” types: “blood”, “urine”, “respiratory”, “gastrointestinal”, and “wound”, assuming isolates come from infection ( *Extended data*, Supplementary Table 1 in *Further Results* ^19^). 14% of susceptibility results had an “other infection site” to these five. Since the most extensive dataset (ATLAS) had age groups instead of age, we used the same age groupings throughout (<=2, 3–12, 13–18, 19–64, 65–84, 85+).

Country information was translated into WHO region using classifications by the World Health Organisation.

### MIC distributions

**Figure 1.**
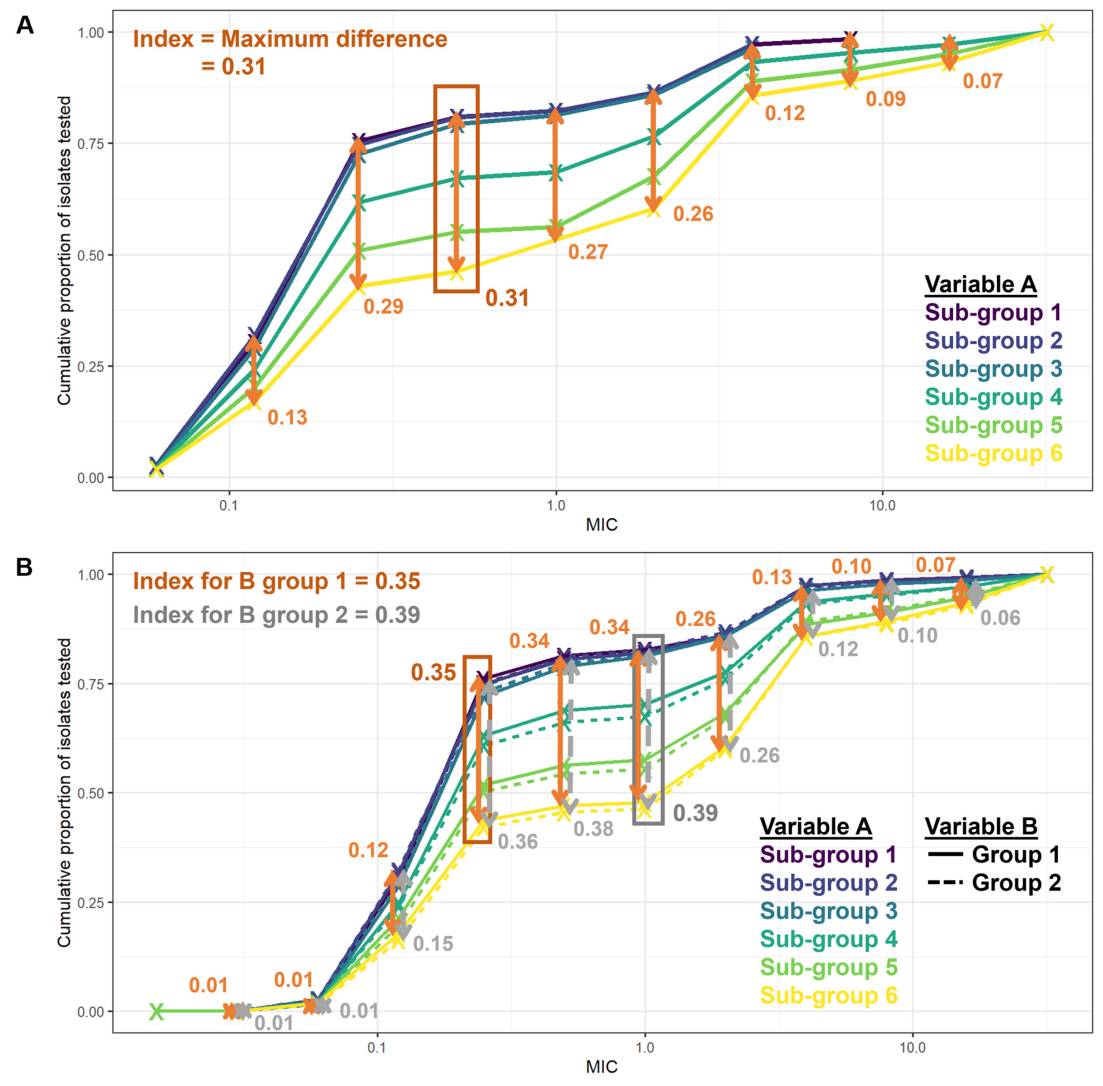
MIC distributions of isolates taken from patients in certain sub-groups and calculation of index values. **A**: Example MIC distributions (line graphs) for a particular bacteria-antibiotic combination, stratified by different levels of variable A (sub-groups 1–6, different colours). The index value for the bacteria-antibiotic combination over variable A, is determined by calculating the largest differences at each MIC value between the curves (gold arrows) and selecting the largest of these differences. **B**: Example MIC distributions (line graphs) for a particular bacteria-antibiotic combination, stratified by different levels of both variable A (sub-groups 1–6, different colours) and variable B (Groups 1–2, different line types). Index values over the variable A can be calculated for different levels of variable B: Group 1 (differences indicated by gold arrows) or Group 2 (silver arrows).

We generated MIC distributions for isolates from each bacteria-antibiotic combination over a chosen key population sub-group based on the information about where the bacteria were isolated (either age group, sex, infection site, infection year, or WHO region). This was achieved by first filtering the central dataset for our bacterial species and antibiotic of interest, before stratifying those results by our chosen sub-group factor of interest, and then visualising the cumulative proportion of isolates found at each MIC value for each level of the key population sub-group factor ( Figure 1A). In some cases, we stratified by more than one chosen key sub-group ( Figure 1B).

### Index value

To measure whether MIC distributions for a given bacterial species and antibiotic showed variation over a key sub-group (e.g. WHO region), we constructed an index value, calculated as the maximum difference between any two cumulative proportions at the same MIC level from different levels of the key sub-group factor ( Figure 1A). Note that while we sometimes stratified MIC distributions by more than one key factor, we only ever measured variability in MIC distributions over one key factor ( Figure 1B).

We defined a bacteria-antibiotic combination to possess notable differences in MIC distributions by a key population sub-group if at least 4 of the largest differences calculated at different MIC levels (before identifying the maximum of these differences as the index value) were greater than 10% (0.1).

### Data analysis

Since we were pooling datasets with an unclear and potentially variable sampling frameworks, we explored variation in the number of MIC values taken over time and how this affected our index calculations. To explore whether higher index values were correlated with bacteria-antibiotic combinations having fewer observations, which would suggest that smaller sample sizes confound the maximum difference in MICs across groupings, we conducted linear regression of index values against sample size for bacteria-antibiotic pairings and patient sub-group factors. To explore the variation over time we explored the number of isolate MICs per year for *S. aureus* and levofloxacin, split by patient age group and sex. Since we hypothesised that differences in the MIC distributions, as indicated by the index value, might be affected by these data issues, we also looked at whether time affected the index value.

### Regression analysis

To determine if differences between sub-groups were confounded by any other sub-groups (e.g. sex by age etc.) we ran a proportional odds regression model. Baseline groups were “adults (age 19–64)” for age group, “female” for sex, “unknown infection site” for infection site, and “Europe” for WHO region.

### Data manipulation, analysis, and visualisation

All data cleaning and analyses were conducted in *R 4.3.1* ^20^ and the code are available at https://github.com/NaomiWaterlow/vivli_paper_kg ^21^. Data can be requested from https://vivli.org/ ^18^.

## Results

### Dataset characteristics

The cleaned and pooled Vivli AMR Register data consisted of 13,180,594 MIC values from four databases (ATLAS, SOAR 201818, KEYSTONE, and GEARS). Of the 401 unique bacterial species, over 278 had fewer than 1000 antibiotic susceptibility results. We focused on the four bacteria with more than 1.4 million results ( *Staphylococcus aureus* (2,448,341) *, Escherichia coli* (1,699,084) *, Pseudomonas aeruginosa* (1,577,954) and *Klebsiella pneumoniae* (1,408,851)) as the next most represented bacteria had less than 750,000.

**Figure 2.**
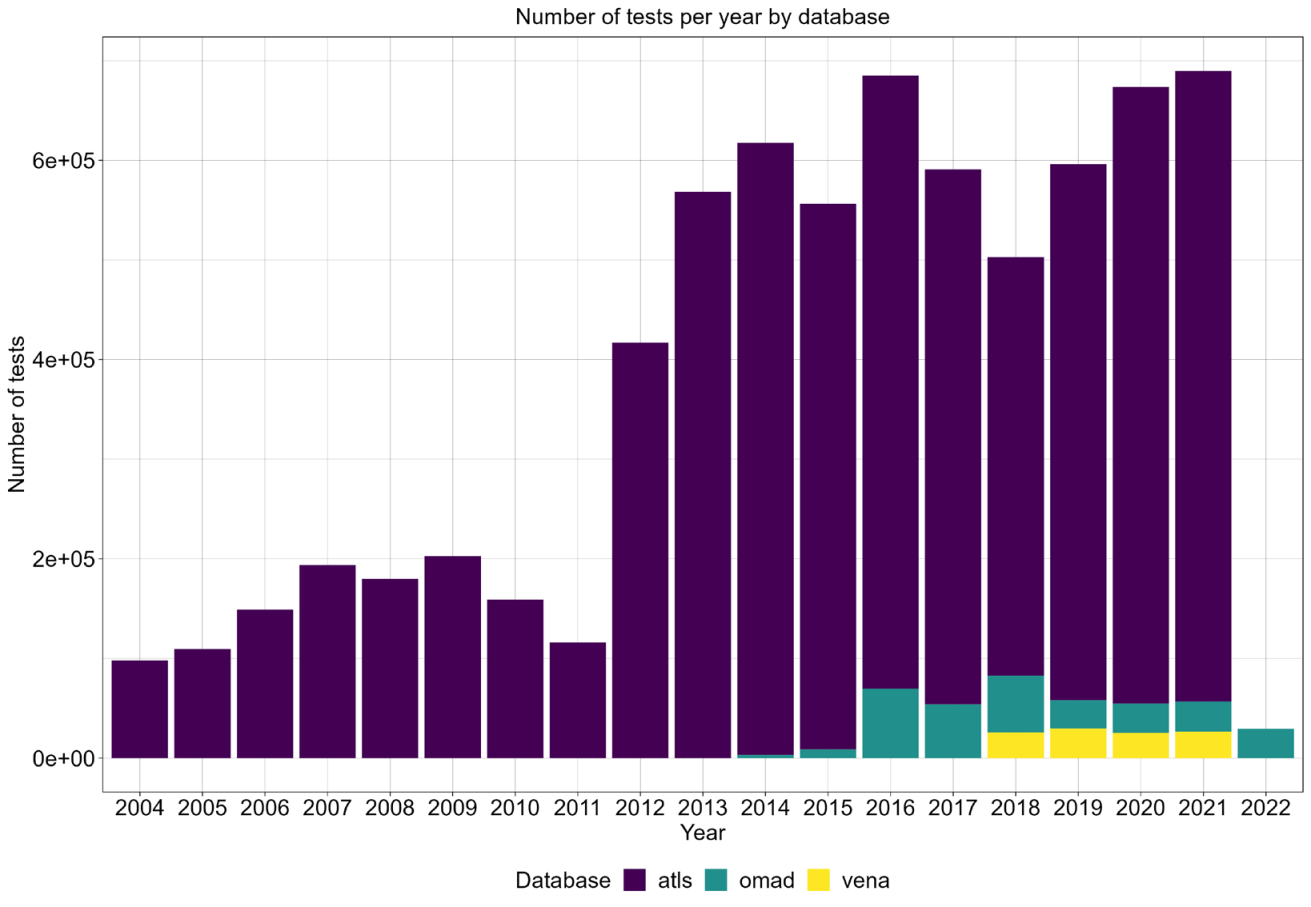
Total number of MIC values from each database in each year included in our final cleaned and pooled dataset: ATLAS (atls, dark blue), KEYSTONE (omad, dark green), GEARS (vena, yellow). Data from SOAR 201818 focussed on bacteria other than our four species of interest and so no results were included from this database.

Our final dataset contained 7,135,070 results, covering 85 countries (grouped under 6 WHO regions) and representing susceptibility to a total of 41 different antibiotics ( *Extended data*, Supplementary Figure 1 in *Further Results* ^19^). The proportions of results within each year increased over time from 2004 to 2022 with a substantial increase in 2012 and most were from ATLAS ( Figure 2). Data from KEYSTONE were incorporated for 2014–2022 and from GEARS for 2018–2021, however these amounted to relatively smaller proportions of the combined dataset. Data from SOAR 201818 focussed on bacteria other than our four species of interest and so no results were incorporated from this database. Overall, ATLAS covered 94.2% of the data, while KEYSTONE and GEARS covered 4.3 and 1.5% respectively ( *Extended data*, Supplementary Figure 2 in *Further Results* ^19^).

The proportion of isolates each year which were *S. aureus* increased substantially in 2012 and remained higher than the other three species until 2018, whereafter it decreased again to comparable levels ( *Extended data*, Supplementary Figure 3 in *Further Results* ^19^). Most tests were obtained from either Europe (47.8%) or the Americas (29.8%) ( *Extended data*, Supplementary Figure 4 in *Further Results* ^19^). In all age groups except the 85+ age group, more male than female samples were included in the datasets with age groups 19–64 and 65–84 being relatively oversampled compared to the other age groups ( *Extended data*, Supplementary Figure 5 in *Further Results* ^19^). Under our groupings for infection site, most isolates were sourced from respiratory and wound infections (26.1 and 24.8% respectively) while only 4.4% were obtained from gastrointestinal infections ( *Extended data*, Supplementary Figure 6 in *Further Results* ^19^).

### Visual analysis of MIC distributions

**Figure 3.**
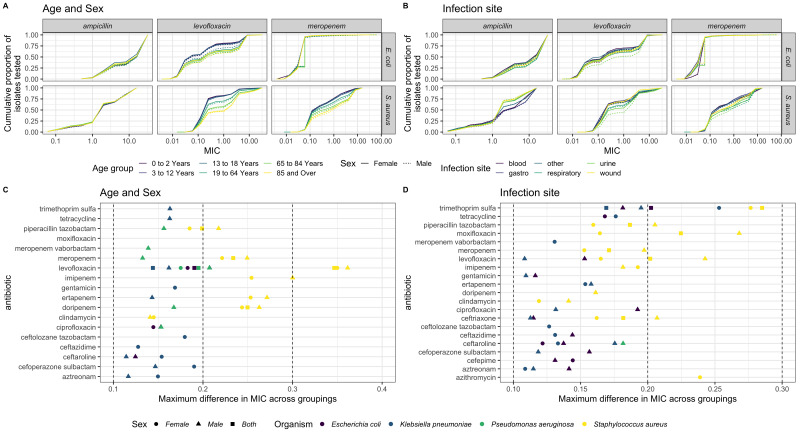
Examples of variation in MIC distribution by age (left) and infection type (right). **A**& **B**: Plots of cumulative sum of isolates tested by MIC for example, bacteria-antibiotic combinations highlight the variation in the MIC distribution by age ( **A**), infection type ( **B**) and sex (line type). Only isolates from the five labelled infection sites were included in the index calculation. Note abbreviations: “gastro = “gastrointestinal”. **C**& **D**: The bacteria-antibiotic pairs with notable differences in MIC distributions (defined as a difference between cumulative population values of more than 0.1 for at least four MIC values).

**Figure 4.**
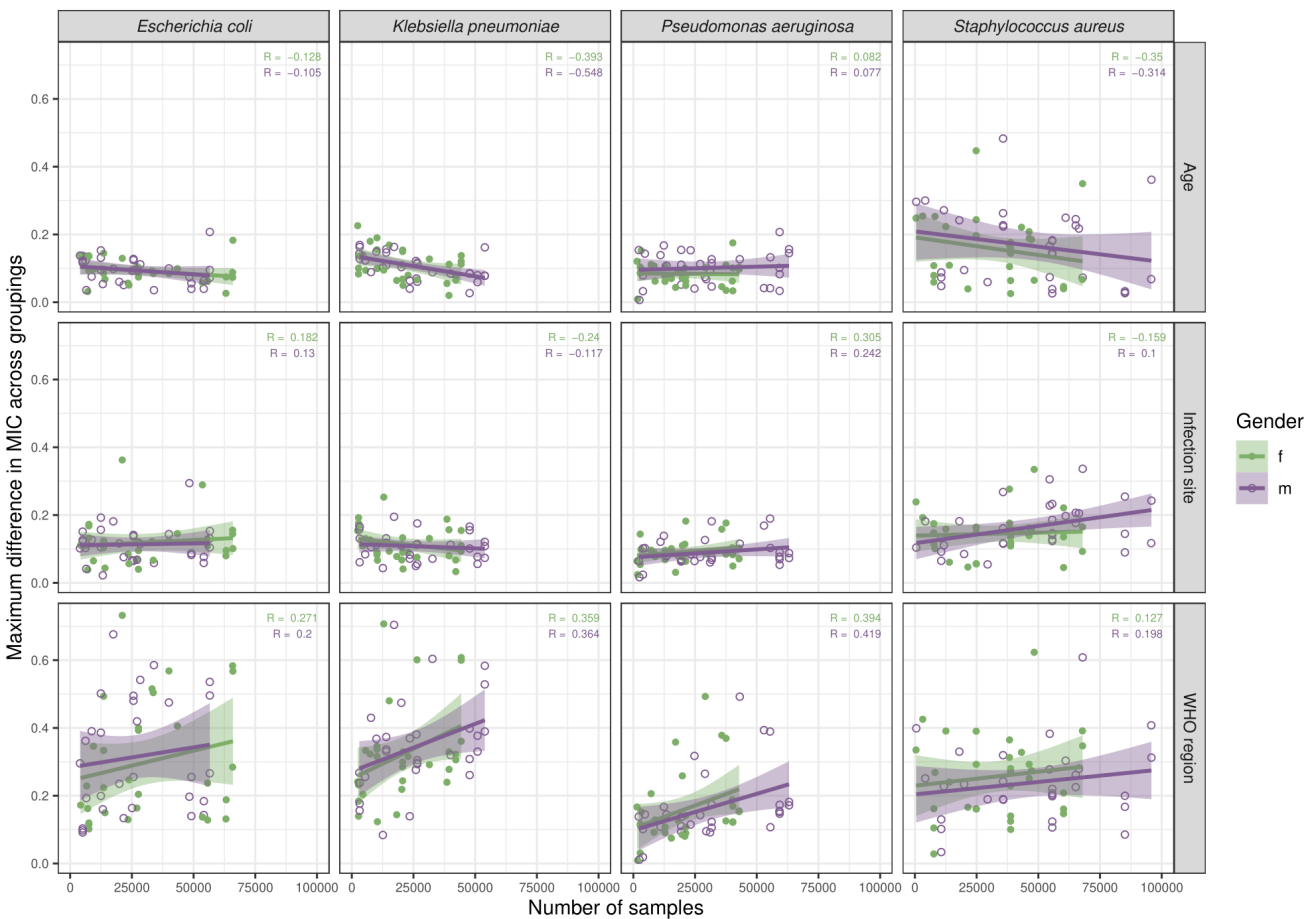
Linear regression reveals no correlation between sample number and index value by grouping factors. Points represent different antibiotics for each species (columns), split by grouping factors (rows) and gender (colour). Correlation coefficients (R) for each gender are provided.

**Figure 5.**
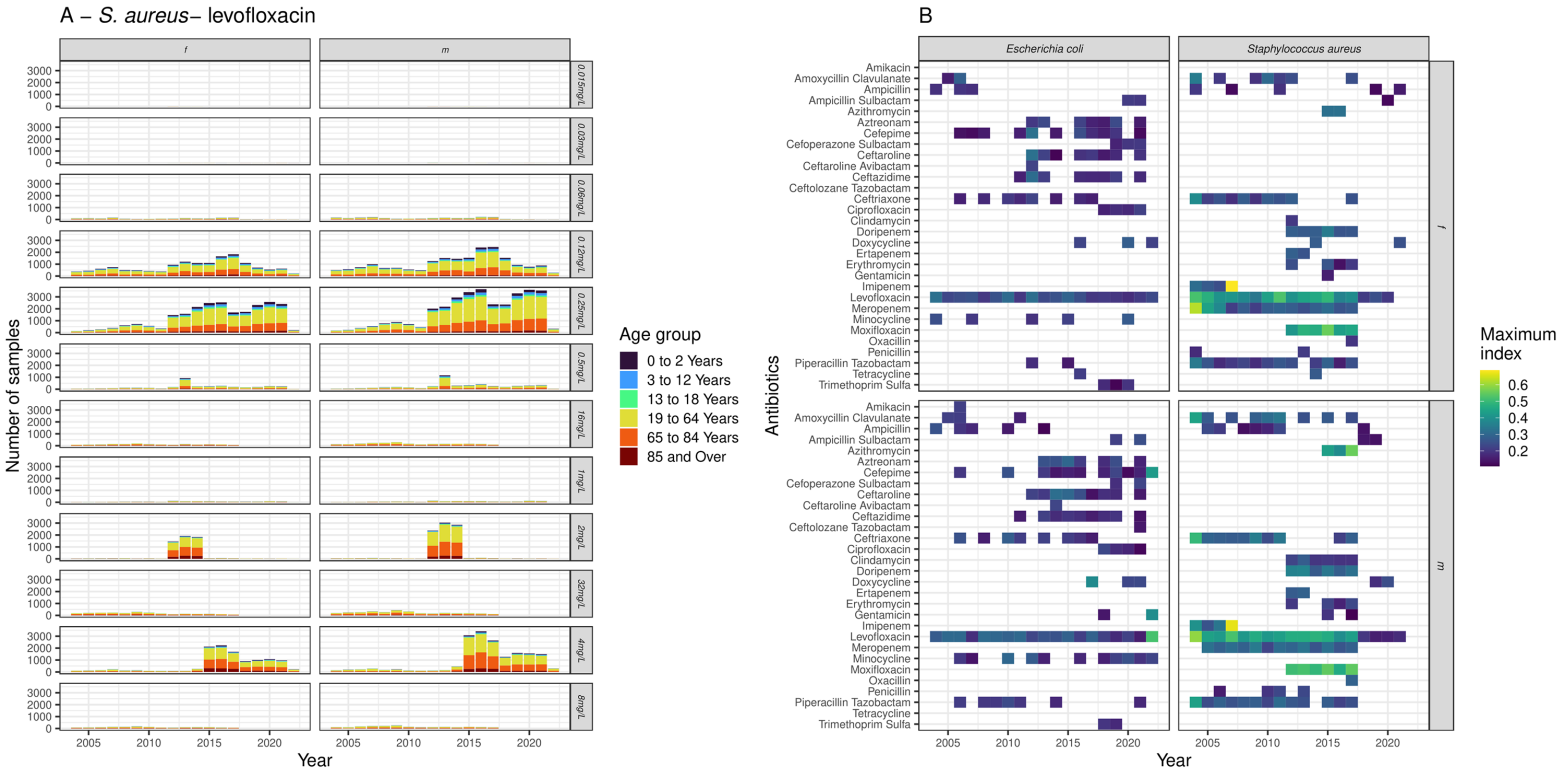
High variability in time is likely linked to changes in data surveillance. **A**: Number of *S. aureus* samples against MIC values for levofloxacin by year, age group and sex. **B**: Tracking the index over time for all bacteria-antibiotic combinations by age group and sex (row) showed a high variation in availability of data and no clear trend in index with time.

**Figure 6.**
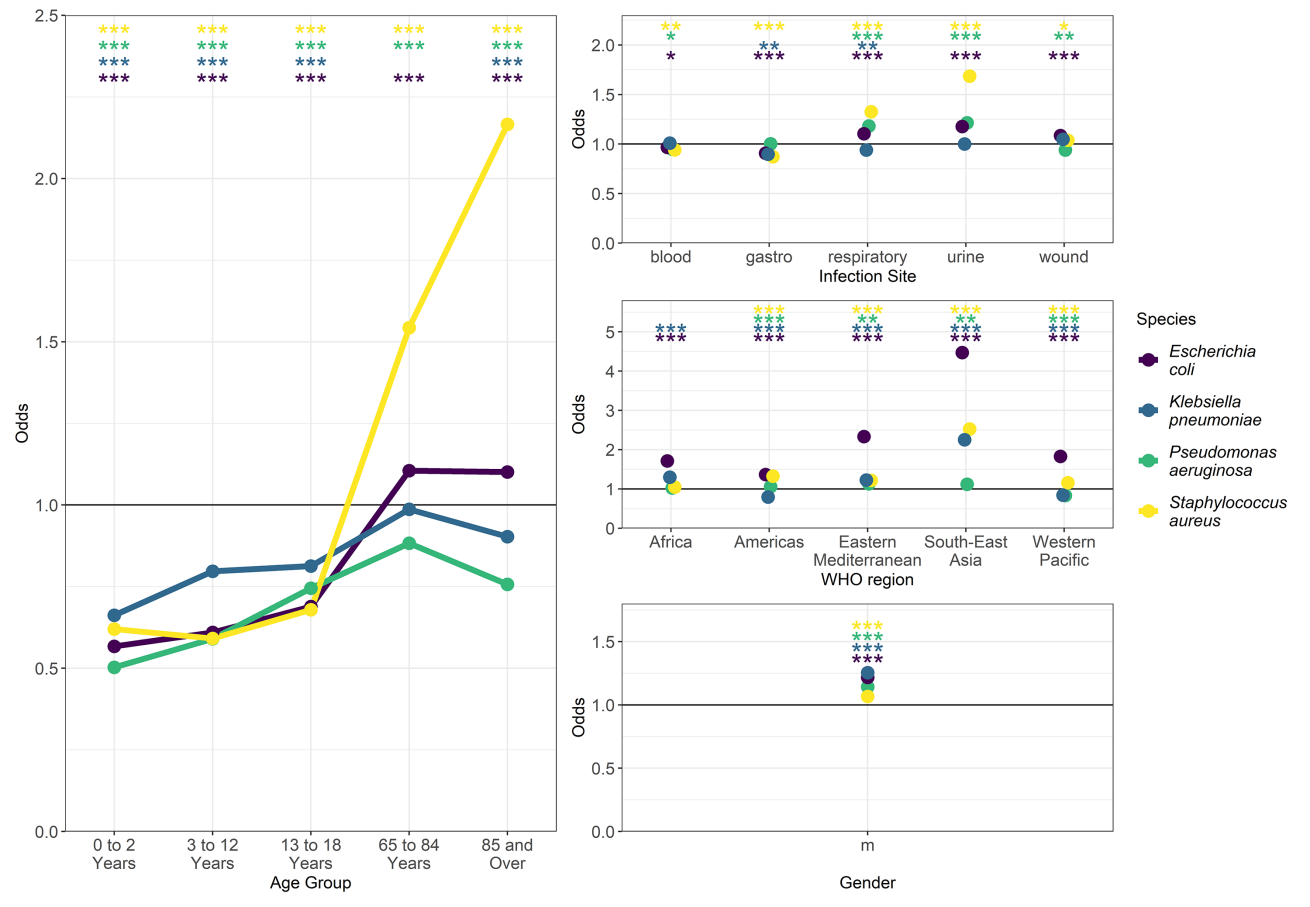
Multivariate regression analysis confirms significant effect of grouping factors on levofloxacin resistance. Odds represent the relative risk of increased MIC compared to the reference group (19 to 64, female, “other” infection site, European). Significance annotation (top) colour represents the species; no text ≥0.05, * <0.05, ** <0.01, *** <0.001.

To identify whether differences in MIC distributions exist between isolates taken from different patient sub-groups, we plotted cumulative MIC distributions for each bacteria-antibiotic combination, stratified by age, sex, infection site, and WHO region ( *Extended data*, Figures 1–24 in *Cumulative plots* ^22^). Visual analysis demonstrated that clear differences in the MIC distribution shape between different sub-groups could be identified. We highlight three antibiotics across two bacteria to demonstrate this ( Figure 3A). In comparison to ampicillin, which does not demonstrate any notable trends by age and sex, levofloxacin demonstrated substantial variation by patient age. This was consistent between sexes, with older age groups (65–84 and 85+) demonstrating an increased proportion of more resistant isolates compared to younger age groups, as shown for *S. aureus* and *E. coli* isolates by the large difference between the MICs of 0.25 and 2mg/L ( Figure 3A).

Differences in isolate resistance between infection sites were also seen, with "urine” infections having higher levofloxacin resistance compared to other infection types. This difference was most pronounced in isolates from men for both *E. coli* and *S. aureus* ( Figure 3B). A clear difference in MIC distributions can additionally be seen for ampicillin resistance in *S. aureus* with a lower proportion of isolates with an MIC above 1mg/L for “blood” and “gastro” isolates vs. “respiratory”, “urine” and “wound”. Substantial differences were also seen in the MIC distributions for many other bacteria-antibiotic combinations across age and sex, infection site, as well as for WHO regions ( *Extended data*, Figure 1–24 in *Cumulative plots* ^22^).

### Analysis of MIC distribution index values

Of those bacteria-antibiotic combinations with high index values (>0.1), indicating large differences in MIC distributions by age and sex, most were in *S. aureus* (50% across all data), followed by *K. pneumoniae* ( Figure 3C). Similarly, we saw bigger differences in the MIC distributions by infection site for *S. aureus*, followed by *K. pneumoniae* and *E. coli,* with few in *P. aeruginosa* ( Figure 3D).

Linear regression of index values against number of MIC tests produced correlation coefficients (R) which varied (age and sex: -0.548 to 0.082; infection site and sex: -0.240 to 0.305) but did not approach 1 nor –1 ( Figure 4). This suggested that sample size did not exert a strong impact on index value, although extremely low values (<100) deviated considerably to produce high index values. Combinations demonstrating <100 samples were therefore excluded from subsequent analyses.

### Variation over time

The number of MIC values varied substantially over time, with the number of results with MICs of 0.12 and 0.24mg/L increasing from 2012 onwards, and the number of samples with an MIC of 4mg/L increasing drastically from 2015 onwards ( Figure 5A).

Tracking the index over time for *E. coli* and *S. aureus* showed no clear trend in index with time suggesting robustness of results and pooling over time ( Figure 5B). A possible contributor to this was that for many combinations there were not enough results for each year to generate an index value. However clear differences in MIC distribution by patient age and sex could be seen for some individual bacteria-antibiotic pairs at different time points. For example, for levofloxacin resistance in *S. aureus,* which showed substantial long-term data by patient age over time ( Figure 5B), there was visible index value fluctuation between 0.3 to 0.6 in both males and females from 2004 to 2017, after which a stable decrease was observed. This suggests that time is a confounding factor.

### Regression analysis to investigate possible confounding interactions

Many of our explored sub-group factors (age, sex, infection site, WHO region, and time) could interact to confound true relationships, particularly in the analyses by age group and time. To overcome this, we conducted regression analyses, demonstrating our results for key bacteria-antibiotic combinations ( Table 1, *Extended data*, Supplementary Tables 2–3 in *Further Results* ^19^). This frequently showed a significant impact of the age of the patient from which the sample was taken on MIC trends, as well as their sex, with varied impact of the infection site but a relatively consistent impact of time.

For example, for levofloxacin resistance in *S. aureus* ( Figure 6, Table 1, first results column), the multivariable regression showed a strong impact of age upon the risk of increased isolate resistance, ranging from odds of 0.62 (ages 3–12) to 2.17 (ages 85+) compared to adults (age 19–64), a small but significant impact of sex (male odds 1.07), and a varied impact of infection site (highest odds in urine). There was also a small but significant decrease in resistance over time (odds 0.98), which is concurrent with the decrease in index observed after 2017 ( Figure 5B, *S.* aureus – levofloxacin).

**Table 1.** Results of proportional odds regression for selected bacteria-antibiotic pairings.

Antibiotic		Levofloxacin								Doripenem				Cefepime			
Bacteria		*Staphylococcus aureus*				*Escherichia coli*				*Pseudomonas aeruginosa*				*Klebsiella pneumoniae*			
Variable		Value	Std. Error	*p* value	Odds	Value	Std. Error	*p* value	Odds	Value	Std. Error	*p* value	Odds	Value	Std. Error	*p* value	Odds
**Age [19 to** **64 years]**	**0 to 2 years**	-0.48	0.02	0.00	0.62	-0.57	0.03	0.00	0.57	-0.50	0.07	0.00	0.61	0.08	0.03	0.00	1.09
	**3 to 12 years**	-0.53	0.02	0.00	0.59	-0.50	0.03	0.00	0.61	-0.46	0.07	0.00	0.63	0.06	0.04	0.17	1.06
	**13 to 18 years**	-0.39	0.03	0.00	0.68	-0.37	0.04	0.00	0.69	-0.42	0.09	0.00	0.66	-0.03	0.05	0.62	0.98
	**65 to 84 years**	0.10	0.01	0.00	1.54	0.10	0.01	0.00	1.11	-0.14	0.03	0.00	0.87	-0.02	0.01	0.20	0.98
	**85 and over**	0.73	0.02	0.00	2.17	0.10	0.02	0.00	1.10	-0.37	0.05	0.00	0.69	-0.13	0.02	0.00	0.88
**Sex** ** [female]**	**male**	0.07	0.01	0.00	1.07	0.20	0.01	0.00	1.22	0.17	0.03	0.00	1.19	0.21	0.01	0.00	1.23
**Infection** ** site ** ** [other]**	**blood**	-0.06	0.02	0.00	0.94	-0.03	0.02	0.05	0.97	0.05	0.07	0.481	1.05	0.12	0.02	0.00	1.13
	**gastrointestinal**	-0.14	0.03	0.00	0.87	-0.10	0.02	0.00	0.91	0.15	0.08	0.05	1.16	-0.15	0.03	0.00	0.86
	**respiratory**	0.28	0.02	0.00	1.33	0.10	0.02	0.00	1.10	0.18	0.04	0.00	1.19	-0.09	0.02	0.00	0.92
	**urine**	0.52	0.03	0.00	1.69	0.16	0.02	0.00	1.18	0.03	0.05	0.509	1.03	-0.09	0.02	0.00	0.92
	**wound**	0.04	0.02	0.02	1.04	0.08	0.02	0.00	1.09	-0.15	0.04	0.00	0.86	-0.07	0.02	0.00	0.93
**WHO** ** region** ** [Europe]**	**Africa**	0.05	0.04	0.19	1.05	0.54	0.04	0.00	1.71	0.04	0.07	0.61	1.04	0.51	0.04	0.00	1.67
	**Americas**	0.28	0.01	0.00	1.32	0.31	0.01	0.00	1.37	0.13	0.03	0.00	1.14	-0.25	0.01	0.00	0.78
	**Eastern ** **Mediterranean**	0.20	0.03	0.00	1.22	0.85	0.04	0.00	2.33	0.38	0.08	0.00	1.46	0.41	0.04	0.00	1.50
	**South-East ** **Asian**	0.93	0.03	0.00	2.53	1.50	0.04	0.00	4.47	0.13	0.08	0.11	1.13	0.86	0.04	0.00	2.37
	**Western Pacific**	0.15	0.01	0.00	1.16	0.60	0.02	0.00	1.83	-0.15	0.03	0.00	0.86	-0.75	0.02	0.00	0.47
**Year**		-0.02	0.00	0.00	0.98	0.09	0.00	0.00	1.09	0.02	0.01	0.01	1.02	-0.05	0.00	0.00	0.95

*Significant p-values (<0.001) are shown in red. Reference groups for each variable are shown in square brackets.*

Age had a variable impact on the risk of increased isolate resistance for other antibiotic-bacteria combinations. For example, all other groups had a decreased odds compared to the comparator age group for doripenem resistance in *P. aeruginosa* ( *Table 1*, third results column, *Extended data*, Supplementary Figure 7 in *Further Results* ^19^), demonstrating a lower risk of resistance in younger and older age groups *.* For cefepime resistance in *K. pneumoniae,* in contrast to levofloxacin resistance in *S. aureus*, the youngest age group (ages 0–2) showed an increased odds (odds 1.09) ( *Table 1*, fourth results column, *Extended data*, Supplementary Figure 8 in *Further Results* ^19^), whereas the oldest showed a slight decrease (0.88). Results for sex showed that males were always linked (in these examples) with higher MIC values ( Figure 6, Table 1). Urinary infections frequently had a higher MIC compared to other groups, but not consistently; similarly, resistance was not always shown to decrease over time ( *Table 1*, second and third results column).

## Discussion

MIC distributions have been used to explore differences in resistance levels between countries and over time. Here we show that they can be used to further explore differences between patient sub-groups (e.g. age groups). For many bacteria-antibiotic combinations, we found substantial differences in MIC distributions between patient sub-groups, identified and confirmed through index value calculation and data visualisation. All four analysed bacteria (those with the largest numbers of MIC results) showed substantial variation in MIC distributions by age and infection site for multiple antibiotics.

Most notably, we found large variation between the MIC distributions of *S. aureus*, which we highlight for the fluoroquinolone levofloxacin across age and infection site. This could be linked to the limited use of quinolones in children due to safety concerns ^23^, but then we might expect a step change between isolates taken from children and adults, not the observed continual differential increase with age. Levofloxacin is not usually used to treat *S. aureus* infections, so whilst these patterns may not be a priority for clinical care, they reveal patterns that point to either transmission or selection variation by age. Patient age group differences were also seen in other bacteria-antibiotic pairings, for example the increased risk of cefepime resistance in *K. pneumoniae* isolated from babies could reflect the burden of *K. pneumoniae* in neonatal intensive care units ^24^ where a recent Shanghai case study found *K. pneumoniae* multidrug resistance, including to cefepime, to be substantial ^25^. These results could be used to design age-structured stewardship patterns or provide better understanding of infection pathways.

We found that the differences in resistance were robust to confounding, with some broad trends that MIC values tended to be higher in isolates taken from urine, males, those of an older age and South-East Asia. These results are supported by the literature ^15, 26, 27^, but are over-simplifications due to the lack of clarity around the sampling framework for these isolates.

For our analysis we combined multiple large multinational datasets in the longitudinal Vivli AMR register. These varied both in terms of which isolates were tested (we assumed for simplicity these were all infecting rather than colonising isolates) and the way testing was conducted (e.g. to calculate MIC) ^28– 33^. This is a major limitation of routine surveillance and limits the applicability of our results and usage of the Vivli AMR register. We also made the simplifying assumption that we could remove character symbols (e.g. “<” and “>=”) from MIC measurements. There will likely also be strong sampling biases in the data that we are unable to account for, due to the different aims for which the data were originally collected. Using multiple Vivli AMR register datasets, instead of focusing on one, helped us to counter this bias to an extent. Overall, however, our analyses point to a new dimension for which existing routine AMR data can be harnessed to understand differences between patient groups and to test hypotheses.

A key step in overcoming these limitations would be to apply our methodology to locally collected data and verify if the patterns seen in these global data hold at the local level. Exploring MIC distribution differences using the Vivli AMR register datasets has enabled us to demonstrate population sub-group differences and to develop a framework that should now be used to complement local analyses. By providing a baseline, these open data are a key resource for AMR researchers, which do not require sometimes difficult negotiations with clinical settings and added data curation burden. At a local level, comparisons between MIC distributions and those in the Vivli AMR register data could point to transmission source differences (e.g. between wards) or to where AMR evolution is accelerated. Future work could apply this methodology to MIC data from a hospital for example, using the differences in MIC distributions to point infection control interventions to specific wards or patient groups.

Due to the averaging of AMR data down to discrete, clinical decision-making thresholds, there has been a lack of analysis at the ecological level of AMR diversity. In this project, we exploit the data available through the Vivli AMR register to take the innovative step of exploring MIC distributions by different patient sub-groups. We demonstrated that for certain bacteria, different strains are likely circulating in different age groups and causing different infections. This work is generalisable as it could be applied to any user-defined groupings, e.g. hospital setting and could be used in any setting where MIC or phenotypic data on levels of resistance is being generated. It could be used to explore MIC distribution variation to gain insight into phenotypic variation and hence variation in transmission and selection across wards, hospitals or at the national level. The impact would be to exploit the differences or similarities in MIC distributions to say where and when interventions could impact resistance evolution, antibiotic selection or transmission. This could be differential targeting of interventions by subgroups or differential antibiotic stewardship guidelines.

## Ethics and consent

Ethical approval and consent were not required.

## Data Availability

This publication is based on research using data from Johnson & Johnson, Venatorx, Paratek, Pfizer, GSK and Shionogi, obtained through https://amr.vivli.org. Analysis code available from: https://github.com/NaomiWaterlow/vivli_paper_kg ^21^. Archived analysis code at time of publication: https://zenodo.org/doi/10.5281/zenodo.11042875 ^34^. License: Code is available under the terms of the Creative Commons Zero "No rights reserved" data waiver (CC0 1.0 Public domain dedication). Figshare: Extended Data - Further Results. https://doi.org/10.6084/m9.figshare.25417615 ^19^. This project contains further information on the generation of our combined dataset (Supplementary Table 1), as well as additional analysis of the dataset characteristics (Supplementary Figures 1–6), and additional subgroup multivariate analysis figures (Supplementary Figures 7–8) and tables (Supplementary Tables 2–3). Figshare: Extended Data – Cumulative Plots. https://doi.org/10.6084/m9.figshare.25418230 ^22^. This project contains all of the cumulative MIC distribution plots for our four chosen key pathogens and all antibiotics, analysed across patient age group, infection site, WHO region, and sex (Figures 1–24). Extended data are available under the terms of the Creative Commons Attribution 4.0 International license (CC-BY 4.0).
