# Supplementary material for: MIC distribution analysis identifies differences in AMR between population sub-groups
Source: Wellcome Open Res. 2024 May 9;9:244. [Version 1] doi: 10.12688/wellcomeopenres.21269.1 (PMC11306957; doi:10.12688/wellcomeopenres.21269.1)
Supplement: Peer review history

# Reviewer report: Reviewer response for version 1

**DOI:** 10.21956/wellcomeopenres.23524.r87258

**Published:** 2024-08-07

**Authors:** Alessandro Gerada

**Affiliations:** 1 University of Liverpool, Liverpool, England, UK

Thank you for the invitation to review this manuscript by Wildfire et al, titled “MIC distribution analysis identifies differences in AMR between population sub-groups”. This is a well written and presented manuscript, which I enjoyed reading. My comments below are relatively minor. My peer review was written independently of other reviewer comments.

**General comments:**

 The use of population sub-groups features heavily within the manuscript. I think the subgroups remain too abstract throughout and could benefit from some details on how the subgroups where determined, and the general characteristics of each group. I may have missed this in the text or supplementary material.

 The use of antimicrobial is preferable to antibiotic.

 Suggest consistent terminology use regarding sex and gender (both seem to be used interchangeably within the manuscript).

 Use of term samples instead of isolates.

 The most striking differences were seen in *S. aureus *(particularly with levofloxacin). However, data on glycopeptide resistance (MRSA) was not included, probably because of insufficient MIC data. It would be interesting to see these results in the supplementary material, if they were analysed.

**Specific comments**

**1) Introduction** - second paragraph. “Minimum inhibitory concentration (MIC), defined as the lowest concentration of a chemical, usually a drug, which pre- vents visible *in vitro *growth of bacteria “.

 Although most MIC measurement methods do rely on visible growth, it would be more accurate to define MIC as the lowest concentration that inhibits growth.

 “Also, MIC is an absolute measurement (though there can be differences by measurement assay) “

 I’m not sure absolute measurement is the correct term, as by its very nature it is highly censored, and usually not reported on a continuous scale. Perhaps it would be more appropriate to say that MIC is more precise than disk susceptibility.

**2) Methods -** second paragraph.

 “Values with a qualifier (e.g. < or ≤) were set at the numeric value in the qualifier (e.g. <8 was set to 8). “

 This data manipulation inevitably adds significant data loss, since the “real” MIC for a value of <8 could be between 0 and 8. However, for a value of 8, the “real” MIC could be between 4 and 8 (for a typical experiment with serial dilutions). Other research has approached this problem by doubling or halving the numeric value at the extremes. Could the authors elaborate on there decision.

 Furthermore, suggest adding units (mg/L) to MIC results.

**3) Methods-** third paragraph

 “assuming isolates come from infection..”

 Was this assumption made because the databases do not specify infection vs colonisation?

**4) Results - **Data characteristics - second paragraph

 “The proportions of results within each year increased over time from 2004 to 2022 with a substantial increase in 2012 and ..“

 Rather than proportions, these appear to be absolute counts of isolates.

**5) Results - **Data characteristics - third paragraph and Figure 2

 The use of “tests” here is confusing as I expect these to be isolates.

**6) Figure 3**

 The use of a log10 scale for the x-axis makes it difficult to follow alongside the text. For example, the text states that older age groups have a large difference between MICs of 0.25 and 2, which is difficult to trace on the plot.

 The legend colours are quite difficult to see without zooming in quite significantly. Can the legend lines be made thicker so that it is clear for example that yellow is the 85 and older?

 Subfigures C and D are well presented and demonstrate the differences nicely.

 I would highly suggest that the axis (both y and x) of subfigures C and D are made to match. Currently, they do not (because of the inclusion of azithromycin in D). This can confuse the reader and make trends difficult to follow. Also, I note that the antimicrobials have been sorted alphabetically. Although this is fine, I think it is worth considering sorting by class in this context, particularly so that quinolones and beta-lactams are next to each other.

**7) Results -** Analysis of MIC distribution index values - second paragraph

 Correlation coefficients (R) are reported but not discussed in methodology. Please can the authors expand on whether these are Pearson correlation coefficients?

**8) Figure 5**

 Subfigure A is a little to busy to interpret without zooming in. Since this is trying to demonstrate changes in time, I don’t think it was necessary to split by sex here. I would suggest removing panels that have no visible data (e.g., 0.015mg/L and 0.03mg/L) so that the figure is easier to read.

 Subfigure B – see previous comments on consideration of grouping by antimicrobial class. Also consider inverting the colour scale so that higher indices have darker colour.

**9) Discussion**

 “but then we might expect a step change between isolates taken from children and adults, not the observed continual differential increase with age…”

 I think it would be interesting to see whether this correlates with increased administration of quinolones by age (in the literature). It may be that these findings are simply because of the low threshold for acquisition of quinolone resistance in *S. aureus*, manifested here as a result of cumulative increased risk of quinolone exposure during individual lifetimes.

 The discussion otherwise makes a good argument for how the analytical approach described in this manuscript could be applied to other, particularly local, datasets.

 The final paragraph does repeat some previous points within the discussion, and could be streamlined somewhat.

Is the work clearly and accurately presented and does it cite the current literature?

Yes

If applicable, is the statistical analysis and its interpretation appropriate?

Partly

Are all the source data underlying the results available to ensure full reproducibility?

Yes

Is the study design appropriate and is the work technically sound?

Yes

Are the conclusions drawn adequately supported by the results?

Yes

Are sufficient details of methods and analysis provided to allow replication by others?

Yes

Reviewer Expertise:

Clinical microbiology, artificial intelligence, machine learning, computer simulation, software development. I have published research on that involved analysis of MIC, and I am the lead software developer of AIgarMIC (an MIC analysis software).

I confirm that I have read this submission and believe that I have an appropriate level of expertise to confirm that it is of an acceptable scientific standard.

# Reviewer report: Reviewer response for version 1

**DOI:** 10.21956/wellcomeopenres.23524.r84836

**Published:** 2024-06-20

**Authors:** Liam P Shaw

**Affiliations:** 1 University of Oxford, Oxford, England, UK

The authors summarise work arising from their entry for a data reuse prize from the Vivli AMR register. They look at MIC distributions and metadata in over 7 million isolates for four species. For a given bacteria-antibiotic combination they define an MIC index over a variable, defined as the maximum difference in MIC between the cumulative proportions for any two subgroups. They show with this metric that are indeed differences in MIC distributions between subgroups and give some examples. It’s a conceptually clear paper and I had no major concerns. Thanks to the authors.

 Some minor comments follow, organised roughly by section. Usual peer review provisos apply: written in haste and I may well have missed things or misunderstood, apologies if so. 

**1. Abstract**

 How could it be used to ‘unveil hidden transmission sources’? I think this could be deleted unless you can substantiate.

 Men having bacteria with more resistance – this surprised me because my understanding was that women suffer disproportionately from AMR burden (driven by pervasive gender inequalities in sanitation, labour). I can see how it's consistent but it might surprise other readers too. Perhaps the authors could comment on this in the discussion.  

**2. Introduction / Methods**

 The motivation is good. This is perhaps pedantic of me, but I don’t think it’s right that we can’t optimally target interventions because we don’t know whether antimicrobial use or transmission causes (perhaps clearer than ‘drives’) more infections than the other. What we really care about is the rough contribution of each. If we knew antimicrobial use caused fewer infections, then that could still be the right area to target first if it was easier to intervene. You could just say that ‘We lack knowledge about optimal interventions, with many stewardship interventions failing to impact AMR rates. Evidence suggests…’

 ‘historical exploration of MIC data’ – ‘exploration of historical MIC data’? Not sure whether the exploration or the data is historical. If the former, then ‘previous’ is clearer.

 Index value: later you call it ‘Maximum difference in MIC acros groupings’ (Fig 3 axis). Maybe it’s useful to repeat that here. I don’t understand the rationale for the ‘notable differences’ metric. If it’s arbitrary please just say so. Might be helpful to say ‘Under this definition, the % of bacteria-antibiotic combinations with notable differences for each variable were: sex (X%), WHO region (X%)….’­

**3. Results**

 ‘SOAR 201818’ is a typo

 Fig 3 – interesting display of the data and I appreciated the choice of three antibiotics with different patterns.

 Fig 4 – the statistical explanation here is wrong. Correlation coefficients do not emerge from performing a linear regression. I know the authors did perform linear regression (I guess: stat_smooth, method=lm) but the R-value shown doesn’t come from that regression. The regression would give you the coefficient of the slope, the R-value is the strength of that linear relationship. R not approaching 1 or -1 doesn’t mean there is no correlation. Though I do agree just from­ eyeballing the slopes and errors it’s probably right that there is no correlation with no. of samples (p<0.05) in any subpanel.

 Fig 5 – highlighting this variability in time is valuable.

 Fig. 6 - The finding of *S. aureus-*levofloxacin showing substantial variation across age and infection site is interesting and a nice example of what looking at MIC distributions can show. I think it’s worth looking in the literature for this as you did for K. pneumoniae: studies exist confirming different population structure of *S. aureus* with age  Sangvik et al. 2011 (Ref 1), Said et al. 2023 (ref 2). I suspect this is driven a lot by the difference between community-acquired and hospital-acquired *S. aureus*, on which there is a big literature too.

**A few other minor comments**

 Wherever ‘samples’ is used (outside of 'sample size') - I believe this should be ‘isolates’.

 The source definitions: I think these look ok except for ‘skin’ being ‘wound’ (!). I presume it only affects a small number of samples so I’m not asking for the entire­ analysis to be rerun, but that was one where I wasn’t convinced that the grouping makes sense.

   

 Why was it necessary to assume these were all infecting rather than colonising isolates? I don’t see anywhere that assumption is really used – you just assume that they can be treated as one in your analysis. Seems you could delete. 

 Ignoring the character symbols in MICs: be aware there is work on modelling MICs accounting for censoring e.g. van de Kassteele et al. 2012 (Ref 3) but I agree it’s fine here to just ignore.

 It might be worth stressing somewhere (in dicsussion or in introduction) is that if all else is equal, the MIC distribution for a collection of isolates comes from the genetic diversity of the strains; therefore, differences in MIC distribution between subgroups should be indicative of different strains (or a different balance of strains) circulating in those subgroups. (Where ‘strain’ might also be ‘the same strain but with the sole difference it is resistant to X’.) I got the feeling this was something you were implicitly assuming but don’t remember an explicit framing of it.

 On local implementation, some thoughts: I would guess this might be done anecdotally/qualitatively anyway? e.g. Doctors noticing that they have lots of isolates with certain profiles in older people. It would be nice to give an example of a more local implementation from the data, but I know this dataset isn't the right one for that due to its granularity. 

 The github: if possible, please write a short description in the README of what the numbered scripts do so other people can more easily use it.

Is the work clearly and accurately presented and does it cite the current literature?

Yes

If applicable, is the statistical analysis and its interpretation appropriate?

Partly

Are all the source data underlying the results available to ensure full reproducibility?

Yes

Is the study design appropriate and is the work technically sound?

Yes

Are the conclusions drawn adequately supported by the results?

Yes

Are sufficient details of methods and analysis provided to allow replication by others?

Yes

Reviewer Expertise:

Computational biology and antimicrobial resistance. I previously published a paper looking at MIC measurements in the ATLAS dataset: https://doi.org/10.12688/wellcomeopenres.15304.3

I confirm that I have read this submission and believe that I have an appropriate level of expertise to confirm that it is of an acceptable scientific standard.

## References

[1] : Age- and gender-associated Staphylococcus aureus spa types found among nasal carriers in a general population: the Tromso Staph and Skin Study. *J Clin Microbiol* .2011;49(12) : 10.1128/JCM.05290-11 4213-8 10.1128/JCM.05290-11 21998436 PMC3232966

[2] : A Sequalae of Lineage Divergence in Staphylococcus aureus from Community-Acquired Patterns in Youth to Hospital-Associated Profiles in Seniors Implied Age-Specific Host-Selection from a Common Ancestor. *Diagnostics (Basel)* .2023;13(5) : 10.3390/diagnostics13050819 10.3390/diagnostics13050819 36899963 PMC10001379

[3] : New statistical technique for analyzing MIC-based susceptibility data. *Antimicrob Agents Chemother* .2012;56(3) : 10.1128/AAC.05777-11 1557-63 10.1128/AAC.05777-11 22232288 PMC3294928
